# Prescribing practices using WHO prescribing indicators and factors associated with antibiotic prescribing in six community pharmacies in Asmara, Eritrea: a cross-sectional study

**DOI:** 10.1186/s13756-019-0620-5

**Published:** 2019-10-22

**Authors:** Nebyu Daniel Amaha, Dawit G. Weldemariam, Nuru Abdu, Eyasu H. Tesfamariam

**Affiliations:** 1 School of Pharmacy, Asmara College of Health Sciences, P.O. Box 8566, Asmara, Eritrea; 2 Pharmacy, Hazhaz Hospital, Asmara, Eritrea; 3 Department of Medical Sciences, Orotta College of Medicine and Health Sciences, Asmara, Eritrea; 4 College of Science, Eritrean Institute of Technology, Mai Nefhi, Eritrea

**Keywords:** Rational medicine use, Predictors of antibiotic prescribing, Community pharmacy, Antibiotic resistance, Eritrea, WHO prescribing indicators

## Abstract

**Background:**

Antibiotics require more prudent prescribing, dispensing and administration than other medicines because these medicines are at a greater risk of antimicrobial resistance (AMR). Studying the current medicine use practices and factors affecting the prescribing of an antibiotic would help decision makers to draft policies that would enable a more rational use of medicines.

**Methods:**

A prospective, descriptive, and cross-sectional study was conducted to assess the current prescribing practices including antibiotics use in six community pharmacies in Asmara. A total of 600 encounters were reviewed using the WHO core prescribing indicators between May 5 and May 12, 2019 using stratified random sampling technique. Descriptive statistics and logistic regression were employed using IBM SPSS® (version 22).

**Results:**

The average number of medicines per prescription was 1.76 and 83.14% of the medicines were prescribed using generic names while 98.39% of the medicines were from the National Essential Medicines List (NEML). The percentage of prescriptions containing antibiotics was 53%. The number of encounters containing injections was 7.8%. Patient age, gender and number of medicines prescribed were significantly associated with antibiotic prescribing at bivariate and multivariable models. Subjects under the age of 15 were approximately three times more likely to be prescribed antibiotic compared to subjects whose age is 65 and above (Adjusted Odds Ratio (AOR): 2.93, 95%CI: 1.71–5). Similarly, males were more likely to be prescribed antibiotic than females (AOR: 1.57, 95%CI: 1.10–2.24). Subjects to whom three to four medicines prescribed were two times more likely to be prescribed an antibiotic compared to those who were to be prescribed one to two medicines per encounter (AOR: 2.17, 95%CI: 1.35–3.5). A one-unit increase in the number of medicines increased the odds of antibiotic prescribing increased by 2.02 units (COR: 2.02; 95%CI: 1.62–2.52).

**Conclusions:**

This study found that the percentage of antibiotics being prescribed at the community pharmacies in Asmara was 53% which deviated significantly from the WHO recommended values (20–26.8%). Furthermore, the percentage of encounters with an injection was 7.8% lower than the WHO value of 13.4–24.0%. Patients’ age, gender and number of medicines were significantly associated with antibiotic prescribing.

## Background

The World Health Organization (WHO) defines rational use of medicines as giving the right medicine, for the right patient, at the right dose, for the right duration and at the right (lowest) cost to them and their community [1]. Different studies have shown that as much as half of prescribed medicines are inappropriately prescribed or dispensed. Moreover the association between an increased misuse of antibiotics and emergence and spread of resistant microorganisms has been confirmed by many studies [2–8]. Inappropriate prescribing of antibiotics, i.e. prescribing an antibiotic when no antibiotic is needed or giving the wrong antibiotic at inappropriate dose for an inappropriate duration, has been pointed out as one of the factors leading to the misuse of antibiotics. Globally, the inappropriate use of antibiotics in primary care and hospital settings is a major contributing factor to the spread of antimicrobial resistance (AMR). WHO has classified AMR as a public health threat of growing concern in need of immediate attention [9–11]. Then it is not surprising that currently more than 700, 000 deaths per year are in some way attributed to AMR and this is projected to reach 10 million lives and cost 100 trillion USD by 2050 [12].

Judicious use of antibiotics in the ambulatory setting contributes to the rational use of antibiotics as a whole because antibiotic consumption in the community setting is an important part of the general antibiotic consumption. Outpatient pharmacies are good starting places where information regarding antibiotic use in the community could be gleaned from. According to the Center for Diseases Control (CDC), 30% of all antibiotics prescribed in outpatient clinics in the United States are unnecessary. Even when antibiotics are needed, states the study, prescribers are often inclined to prescribe medicines that may be less effective and carry more risk over the “first-line” medicines recommended by essential medicines list (EML) or national guidelines [13].

Low income countries (LIC) suffer from an increased burden of infectious diseases, shortage of medicines and competent healthcare professionals. Prescribers when faced with shortage of medicines are more likely to prescribe irrationally. AMR rates are higher in LIC as compared to high income countries (HIC) and irrational medicine use is higher in developing countries than the developed world [3, 14, 15].

There are many factors which could affect whether a patient is prescribed an antibiotic or not. Gender and age could determine the extent of antibiotic prescribing. A study done in the UK reported that females received 43% more antibiotics than males, and adult females were more likely to receive an antibiotic than either children or elderly [16].

WHO in collaboration with the International Network of Rational Medicine Use (INRDU) has developed a group of indicators to assess the use of antibiotics in health facilities. The three main indicators are prescribing indicators, facility indicators and patient care indicators. These set of indicators could be used as benchmarks to enable the introduction of antibiotic stewardship programs (ASPs) in different healthcare settings [2, 3, 15]. Many studies have reported using the WHO indicators [17–25]. Studying prescribing patterns of medicines in general and antibiotic in particular aids in identifying irrational prescribing behaviors to make therapy more rational and cost effective [26].

Eritrea, a small developing country in the horn of Africa, is faced with the challenges of irrational medicine use and implementing rational use of medicines. A recent study conducted in the inpatient setting of a tertiary Eritrean hospital reported a high rate of antibiotic use [27]. Thus studying the prescribing behaviors would help in paving a way towards the introduction of ASP in Eritrean hospitals. Many hospitals in the developed world have introduced ASPs to combat the irrational use of antibiotics.

The introduction of ASP requires the study of antibiotics prescribing behavior in inpatient and outpatient settings as a benchmarking tool before its implementation. Given the notable difference in socio-economic standing among different countries, the prescribing indicators of developing countries deviate significantly from the WHO reference targets [2]. It is our hope that this study would serve as a starter for a much broader study of antibiotics and the introduction of ASPs in Eritrean hospitals.

To the best of our knowledge there is no such published research conducted in Eritrea and thus the status of prescribing behavior is unknown. The aim of the study was therefore to assess the current prescribing behavior using WHO core medicine prescribing indicators and to determine the association of patient gender, age and total number of medicines prescribed with antibiotic prescribing.

## Methods

### Study design

A descriptive and cross-sectional study with a quantitative approach was conducted prospectively to assess prescribing behavior and factors associated with antibiotic prescribing.

### Study setting

Eritrea has a total of 49 community pharmacies and 39 drug shops. Out of the 49 community pharmacies, 13 are governmentally owned. Six of the governmentally owned community pharmacies are found in Asmara. Two of them are found inside the national referral hospitals: Orotta and Halibet. Besides, the majority of patients who cannot find their medicines inside the Out-patient department (OPD) pharmacies of hospitals get their medicines from these six governmentally owned community pharmacies located in Asmara because of the better availability and affordability of medicines in these pharmacies. The study was carried out in six governmentally owned community pharmacies in Asmara, the capital city of Eritrea with a population of 427, 183 according to a 2017 report [28]. A “prescriber” in the Eritrean context and this article includes Medical Doctors, Nurses, Associate Nurses, Health Assistants and other lower health cadres could prescribe based on the setting and scarcity of other health professionals.

### Sample size and sampling technique

According to WHO, at least 600 encounters should be included to study current prescribing practices of a facility [29]. A stratified random sampling was used. The six community pharmacies were considered as strata. Within stratum, systematic random sampling was used. The 600 prescriptions were then proportionally allocated. Expected total prescriptions from the six community pharmacies in 6 working days are 4560. By dividing it into 600, every 8th prescription was selected to reach the required sample size.

### Data collection

Data was collected from prescriptions prospectively between May 5 and May 12, 2019 by three pharmacists. All the necessary data required to measure the prescription indicators were then entered into a WHO standard prescription indicator collection form. To avoid ambiguities in the definition of an “antibiotic”, a previously set definition by WHO [29] was employed. By complying with WHO methods and guidelines, data reliability was assured.

### Data processing and statistical analysis

The collected data were double entered on CSPro Software (version 7.0) and exported to Statistical Package for Social Sciences (IBM SPSS Statistics for Windows, Version 22.0. Armonk, NY: IBM Corp.) for statistical analysis. Descriptive summaries of the socio-demographic variables were computed using mean, standard deviation (SD) or median interquartile range (IQR) as appropriate. The association between antibiotic prescribing and its predicting factors was explored using logistic regression. Odds ratio with 95% confidence interval was reported in all logistic regression analyses. All analyses were considered significant when *p* < 0.05.

## Results

A total of 600 prescriptions were analyzed, corresponding to 600 patients, of which 60.3% were female and 38.8% male with a median age of 25 (IQR: 42) (Table 1).

**Table 1:** Socio-demographic characteristics of the study population (*N* = 600)

Patient Characteristics	Frequency	Percent
Age (Md = 25, IQR = 42, Min. = 1, Max. = 90)		
Less than 15	203	35.5
15 to 64	283	49.5
65 or above	86	15
Gender		
Male	233	39.2
Female	362	60.8

A total of 1055 medicines were prescribed. The average number of medicines per prescription was 1.76 (Standard Deviation (SD): 0.85). Most of the medicines were prescribed by generic name (83.14%). Almost all the prescribed medicines (98.39%) were on the Eritrean essential medicines list. Antibiotics were prescribed in 331 encounters (53%). Forty seven prescriptions had injections amounting to 7.8% of encounters (Table 2).

**Table 2:** Prescription indicators using WHO core indicators and Africa Region

Prescribing indicators assessed	Total medicines or encounters	Average or percent	WHO Optimal Value [1]	Standard derived for Africa region [2]
Average number of medicines per encounter	1056	1.76	1.6–1.8	< 2
Percentage of encounter with antibiotics	318	53.0%	20–26.8	45.9
Percentage of encounter with injection	47	7.80%	13.4–24.1	28.4
Percentage of medicines prescribed by generic	878	83.14%	100	65.1
Percentage of medicines from essential medicine list	1039	98.39%	100	89

Of the total number of medicines prescribed, 349 (33.1%) were antibiotics. The most commonly prescribed antibiotics were: amoxicillin (42.1%), ciprofloxacin (20.1%), co-trimoxazole (9.5%) and amoxicillin-clavulanate (4.9%). Medicines most commonly co-prescribed with an antibiotic were: paracetamol (*n* = 106/326), Oral Rehydration Salt (ORS) (*n* = 31/326), omeprazole (*n* = 22/326) and ibuprofen (*n* = 21/326).

The WHO has developed a classification system called AWaRe which stands for Access, Watch and Reserve [30]. According to this classification antibiotics have been classified into three groups based on whether they need to be generally accessed (Access Group) or carefully watched (Watch) group or only reserved for special situations (Reserve Group). The following table shows the percentage of individual antibiotics and their category (Table 3).

**Table 3:** Classification of the antibiotic types using the AWaRe methodology [30]

AWaRe Classification		
Access (%)	Watch (%)	Reserve (%)
Amoxicillin (59.3)	Ciprofloxacin (90.9)	–
Co-trimoxazole (13.3)	Clarithromycin (9.1)	
Metronidazole (6.9)		
Amoxicillin/Clavulanate (6.9)		
Doxycycline (6.5)		
Chloramphenicol (2.4)		
Others (4.7)^a^		

^a^ Cloxacillin, Penicillin,Tinidazole, Gentamycin and Silver sulfadiazine

### Predictors of antibiotic prescribing

Age (*p* < 0.001), gender (*p* = 0.002) and number of medicines prescribed (*p* = 0.025) were significantly associated with antibiotic prescribing at the bivariate level (Table 4).

**Table 4:** Predictors of antibiotic prescribing

Variable	Was Antibiotic Prescribed?		Bivariate analysis	
	No n (%)	Yes n (%)	COR (95% CI)	*p*-value*
Age				< 0.001
< 15	65 (24.4)	138 (45.1)	2.68 (1.60–4.50)	< 0.001
15 to 64	153 (57.5)	130 (42.5)	1.07 (0.66–1.74)	0.775
65 and above	48 (18)	38 (12.4)	Ref.	
Gender				
Male	91 (32.5)	142 (45.1)	1.71 (1.22–2.38)	0.002
Female	189 (67.5)	173 (54.9)	Ref.	
No of medicines prescribed				0.025
1 to 2	249 (88.3)	248 (78.0)	Ref.	
3 to 4	32 (11.3)	65 (20.4)	2.04 (1.29–3.23)	0.002
5 or more	1 (0.4)	5 (1.6)	5.02 (0.58–43.28)	0.142

*COR* Crude Odds Ratio, *CI* Confidence Interval, *p < 0.05 was considered significant

The full model containing all predictors was statistically significant, χ2 (5, *N* = 600) =50.71, *p* < 0.001 indicating that the model was able to distinguish between respondents who had prescribed to antibiotics and did not. Moreover, good fit was observed as per the Hosmer-Lemeshow goodness fit test (χ2 = 3.91, df = 7, *p* = 0.789). The model as a whole explained between 8.5% (Cox and Snell R-squared) and 11.4% (Nagelkerke R-squared) of the variance in antibiotic prescription and correctly classified 63.0% of those who had antibiotic prescription. Sensitivity of the model, percentage of the group with antibiotic prescription that had accurately identified by the model was 58.3%. Moreover, the specificity was 64.2%.

By adjusting potential confounders through multivariate logistic regression, age (*p* < 0.001), gender (*p* = 0.013) and number of medicines prescribed (*p* = 0.014) were still significantly associated with antibiotic prescribing. Subjects under the age of 15 were approximately three times more likely to be prescribed antibiotic compared to subjects whose age is 65 and above (AOR: 2.93, CI: 1.71–5). Similarly, males were more likely to be prescribed antibiotic than females (AOR: 1.57, CI: 1.10–2.24). Subjects to whom three to four medicines prescribed were two times more likely to be prescribed antibiotic compared to those who were to be prescribed one to two medicines per encounter (AOR: 2.17, CI: 1.35–3.5) (Table 5).

**Table 5:** Multivariate analysis of predictors of antibiotics

Variable	Multivariable analysis	
	AOR (95% CI)	*p*-value*
Age		**< 0.001**
< 15	2.93 (1.71–5)	**< 0.001**
15 to 64	1.16 (0.70–1.91)	0.572
65 and above	Ref.	
Gender		
Male	1.57 (1.10–2.24)	**0.013**
Female	Ref.	
No of medicines prescribed		**0.014**
1 to 2	Ref.	
3 to 4	2.17 (1.35–3.5)	**0.001**
5 or more	6.24 (0.7–55.46)	0.1

*AOR* Adjusted Odds Ratio, *CI* Confidence Interval, * *p* < 0.05 was considered significant

There is a significant increase in antibiotic prescribing with an increase in the number of medicines prescribed (*p* < 0.001). As the number of medicines prescribed increases by one unit, the odds of antibiotic prescribing increased by 2.02 units (COR: 2.02; 95%CI: 1.62–2.52).

## Discussion

### Prescribing indicators

In our study the average number of medicines per encounter was 1.76, consistent with the WHO optimal value of 1.6–1.8; which will be henceforward referred to as the standard (Table 2). However, when compared with other studies, it was much lower than 2.8 reported from India [1], 2.34 from Ethiopia [17], and 2.4 from Saudi Arabia [22]. This variation in the number of medicines prescribed per patient could be due to the difference in the study settings and prescribing practices among various medical specialties.

Out of the 600 encounters studied, 53% encounters contained antibiotics (standard 20–26%). This is slightly higher than the 46% for the Africa Region [2] and lower than what other studies have reported from Ethiopia 82.5% [31] and 58.1% [32]. This high percentage of antibiotics being prescribed may be due various reasons. First increased prevalence of infectious diseases in the developing countries results in increased amounts of antibiotics being prescribed, second the high level of routine empirical treatments seen in resource-poor countries. A third reason could be patient pressure on prescribers. A very recent study [33] conducted in Asmara found the rate of self-medication using antibiotics to be 45.1%. This result might show that since patients are highly involved in self-medication using antibiotics, they are more likely to ask prescribers directly or indirectly to prescribe antibiotics. Fourth, allowing other healthcare professionals e.g. health assistants, associate nurses, nurses, health technicians and others to prescribe. In Eritrea one physician serves 18,041 patients, to cope with this shortage of physicians, low level health cadres are licensed to prescribe medicines [34]. As the level of expertise of the prescriber decreases the odds of irrational medicine prescribing is more likely to increase.

We found that in 7.8% of all the encounters at least one injection was included (Table 2). This figure is lower than the 13.4–24.1% WHO standard. Since administration of an injection is associated with risks and injections need trained health care professional for administration, prescribers are advised to prescribe non-parenteral routes of administration whenever possible. Other studies have reported higher rates of injections being prescribed in Ethiopia 11.2% [31], 26.5% [35] and 38% [32]. These studies from Ethiopia were conducted in hospital pharmacies which could somehow account for the increased percentage of injections being prescribed.

Prescribing using generic name permits dispensers to substitute therapeutic equivalents when one particular brand is not available and generic medicines are cheaper which ensures better availability in the market. Generic name prescribing was 83.1%, lower than the recommended 100% (Table 2). Better generic prescribing studies have been reported from Ethiopia [31] at 97%, Iran 95% [3], and Ghana 93% [36]. This could be due to the old prescribing habits of prescribers, familiarity with brand names. Based on our results, generic name prescribing is something which needs to be addressed in Asmara healthcare settings. Educational activities targeting prescribers should be held to promote generic name prescribing and medical students should be encouraged to use generic prescribing.

Almost all (98.83%) of the medicines were prescribed from the EML (Table 2), this is similar with a study done in Ethiopia which reported 98.7% [32]; 80.3% [23]. However, our result was much higher than the 28.5% reported by in India [37]. Eritrea follows a centralized procurement system which adheres to the EML; this could in part explain the high percentage of the medicines being prescribed within the EML.

In our study 71.9% (251/349) of the antibiotics were on the Access group while 22.1% (77/349) were in the Watch group and no single antibiotic (0%) was from the Reserve group. And 6% (21/349) of the antibiotics were ungrouped in these three classes. From the Watch group, Ciprofloxacin accounts for 90.1% of the total medicines used in the category. Therefore the use of Ciprofloxacin in the community needs to be carefully monitored.

### Predictors of antibiotic prescribing

This study found significant association of antibiotic prescription with patients’ age, gender, and number of medicines (Table 4). Being under the age of 15 was significantly related with antibiotic prescription. Similar studies from Bangladesh [38], Yemen [39], Cameroon [40] found that patients under the age of 15 received the highest proportion of antibiotics when compared with the other patient groups.

Our study found that males were more likely to receive an antibiotic than females, similar to a study in Bangladesh [38] and in Ethiopia [41]. This is in sharp contrast with a study done by Smith et al. [16], Anong et al. [40] and Streit et al. [42], where they reported that female gender was more significantly associated with antibiotic prescribing. This study showed that prescribing 3 to 4 medicines per encounter was significantly associated with antibiotic prescribing (Table 5). An increase in one unit of medicine increased the odds ratio of using an antibiotic by 2.02, this was similar to a study from Zambia which reported that a one-unit increase increased the odds by 2.7 (*P* < 0.001; OR = 2.68, 95%CI 2.20–3.25) [43].

Since the study was a cross-sectional one, the results might not reflect the seasonal variation of medicine use. Furthermore, the findings of the study were based on the capital city, Asmara, so it might not be generalized to the whole country. Further nationwide studies are needed to get the complete picture of medicine use in the country.

## Conclusion

Our study revealed that the prescribing indicators in the public community pharmacies in Asmara showed deviation from the WHO standard. The percentage of prescriptions which contained an antibiotic was 53% which is double of the optimal values set by WHO (20–26.8%) and the percentage of encounters with an injection was 7.8% lower than the WHO value of 13.4–24.0%. Average number of medicines prescribed per encounter, adherence to EML and percentage of encounters with injection were in compliance the WHO recommended values. Patient age, gender and number of medicines per encounter showed significant association with antibiotic prescribing.

To promote generic prescribing and comply with the INRDU/WHO recommendations, regular updating and wide dissemination of National Standard Treatment Guideline and should be promoted, extensive continuing education programmes targeting prescribers should be introduced. At the hospital level, the Medicines and Therapeutic Committees (MTCs) should be strengthened and new MTCs set up in hospitals where no such committees exist. Based under the supervision and recommendation of MTCs ways of introducing Antibiotic Stewardship Programmes (ASPs) should be diligently sought.

## Data Availability

The data used in this study is available from the corresponding author upon reasonable request.

## Abbreviations

AMR: Antimicrobial Resistance
AOR: Adjusted Odds Ratio
ASP: Antibiotic Stewardship Program
CDC: Center for Disease Control
COR: Crude Odds Ratio
EML: Essential Medicines List
HIC: High Income Country
INRDU: International Network of Rational Medicine Use
LIC: Low Income Country
MTC: Medicines and Therapeutics Committee
RDU: Rational Medicine Use
SPSS: Statistical Package for Social Sciences
WHO: World Health Organization
